# miR-449a regulates insulin signalling by targeting the Notch ligand, Jag1 in skeletal muscle cells

**DOI:** 10.1186/s12964-019-0394-7

**Published:** 2019-07-25

**Authors:** Shagun Poddar, Devesh Kesharwani, Malabika Datta

**Affiliations:** 1 CSIR-Institute of Genomics and Integrative Biology, Mall Road, Delhi, 110 007 India; 2 Academy of Scientific and Innovative Research, CSIR-HRDC, Kamala Nehru Nagar, Ghaziabad, Uttar Pradesh 201002 India

**Keywords:** miR-449a, Notch signalling pathway, Insulin signalling, Skeletal muscle cells, Jag1, Diabetes

## Abstract

**Background:**

miR-449a, an intronic miRNA, is highly down-regulated in the skeletal muscle during diabetes. Its levels are epigenetically regulated by altered acetylation/deacetylation on the promoter that it shares with its host gene, Cdc20b. However, the cellular role of this epigenetically regulated miRNA in the muscle during diabetes is not well understood. Here, we sought to unravel the crosstalk between altered miR-449a expression and impaired skeletal muscle metabolism.

**Methods:**

Predicted targets of miR-449a were extracted using online available target prediction tools. Differentiated C2C12 cells were transfected with the miR-449a mimic and/or its inhibitor and the levels of the target mRNA and protein was evaluated by qRT-PCR and Western Blot analysis. This was validated by luciferase wild type and mutated constructs of the target 3’UTR. Inhibition of Notch signalling was assessed by evaluating the transcript levels of Notch target genes, Hes1 and Hey1 and the status of NICD (Notch Intracellular domain) by immunofluoresence microscopy. Effect of miR-449a on insulin signalling was evaluated by monitoring insulin induced PI3K and AKT phosphorylation and glucose uptake.

**Results:**

Our data demonstrate that in C2C12 skeletal muscle cells, miR-449a binds to the 3’UTR of Jag1, an important Notch ligand, and down-regulates, both its transcript and protein levels. This was, however, prevented in the presence of the miR-449a inhibitor that suggests the specificity of the miRNA effect. This was validated in human primary skeletal muscle cells where miR-449a decreased Jag1 protein levels and this was prevented in the presence of the miR-449a inhibitor. This miR-449a-Jag1 interaction subsequently affects the Notch signalling pathway as was evident by the fact that miR-449a decreased the levels of NICD and consequently, the levels of Notch target genes, Hes1 and Hey1 were significantly inhibited. miR-449a and Notch pathway inhibition using DAPT, significantly increased insulin stimulated PI3K and AKT phosphorylation and these were prevented in the presence of the miR-449a inhibitor.

**Conclusion:**

Our results indicate towards a critical role for miR-449a and its target, Jag1 in regulating Notch signalling and insulin signalling in the skeletal muscle and imply that targeting this axis might hold therapeutic potential for impaired skeletal muscle metabolism during diabetes.

## Background

MicroRNAs (miRNAs) are small (~ 22 nucleotides) endogenous RNA sequences that regulate gene expression by primarily binding to the corresponding target mRNA 3′ UTR sequence and promoting mRNA degradation and/or translational repression. Their expression is controlled mainly at the transcriptional level [1–3] wherein epigenetic modifications play a crucial role in determining their cellular levels [4, 5].

Several reports demonstrate marks for DNA methylation and histone modification on the transcription units of miRNA genes that influence their expression, deregulation of which results in a disease. The contribution of epigenetic mechanisms in altering miRNA levels during colorectal cancer has been reported by Suzuki et al., where 47 miRNAs were altered due to epigenetic silencing in cancerous tissues as compared to normal [6]. Silencing of miR-199a* in several cancer cell lines occurs due to hypermethylation of its gene [7]. This miRNA inhibits the proto-oncogene, MET and its downstream effector, ERK2 and this interaction affects cell proliferation, motility and invasive capabilities of tumor cells.

Other miRNAs like miR-200c/141, 29b/c, 375, 34a, etc. are regulated by DNA methylation and such regulation is critical in different cancers, EMT (Epithelial-Mesenchymal transition) processes and other diseases [8–11]. Members of the miR-29 family are regulated by histone deacetylation and trimethylation. In aggressive lymphomas, miR-29a levels are increased due to decreased recruitment of HDAC3 and PRC2 on its promoter sequence [12] and this consequently increases transcription of miR-29a.

In a previous report, we had demonstrated that in the skeletal muscle during diabetes, elevated HDAC levels and activity regulate miR-449a levels [13]. In-vitro studies demonstrated that the miR-449a (together with its host gene, Cdc20b) promoter harbors core histone acetylation marks that showed increased occupancy of acetylated histones during HDAC inhibition. This increased occupancy possibly facilitates transcription by making the promoter region accessible to the transcription factor complex. Thus, miR-449a is an epigenetically regulated miRNA and its altered levels might be critical in diverse pathological states.

Altered miR-449a levels are implicated in several diseases. miR-449a has been suggested as a tumor suppressor in hepatocellular carcinoma as it inhibits the expression of ADAM 10 (A Disintegrin And Metalloproteinases 10). This negative correlation influences cell proliferation, colony formation, migration and invasion [14]. It suppresses epithelial- mesenchymal transition and metastasis of hepatocellular carcinoma [15]. Such tumor-suppressive function of miR-449a has also been observed in human glioblastoma where the miRNA targets Myc- associated zing finger protein [16] and in prostate cancer where it targets HDAC1 [17]. miR-449a has been identified as a potential novel diagnostic marker for the wingless (WNT) group of medulloblastoma [18].

While miR-449a has been widely implicated in various types of cancers, its and in the skeletal muscle during diabetes remains unknown. Since, we had identified altered levels of miR-449a in the skeletal muscle during diabetes [19] subsequently decoded it to be epigenetically regulated [13], specifically through histone acetylation/deacetylation, we sought to study the roles of this epigenetically regulated miRNA in the skeletal muscle. Our data show that miR-449a targets Jag1, a principal component of the Notch signalling pathway and in doing so, alters the insulin signalling pathway.

## Methods

### Bioinformatic analysis

For miRNA target prediction, three popular public databases, TargetScan (http://www.targetscan.org/), miRanda (http://www.microrna.org/microrna/) and miRDB (http://mirdb.org/miRDB/) were used. A list of consensus target genes that were predicted by all the three tools was analyzed using the KEGG database within Enrichr (http://amp.pharm.mssm.edu/Enrichr/). Biological pathways that were over-represented with a statistically significant probability (adjusted *p* < 0.01) were prioritized.

### Cell culture and transfection

C2C12 mouse myoblast cells were obtained from the National Centre for Cell Science (NCCS), Pune, India and maintained in Dulbecco’s modified Eagle’s medium (DMEM) supplemented with 10% (v/v) heat-inactivated fetal calf serum (Life Technologies, CA, USA) along with 100 units/ml penicillin, 0.1 mg/ml streptomycin (GIBCO, NY, USA) in the presence of 1.5 g/L sodium bicarbonate at 37 °C and 5% CO_2_. At a confluence of 70–80%, the medium was replaced with DMEM supplemented with 2% horse serum (differentiation medium) to promote myoblast differentiation into myotubes that were visible after four days of incubation. Differentiated C2C12 cells were transfected with either the scramble or the miR-449a mimic (10–75 nM) with or without the miR-449a inhibitor (10 nM) (Dharmacon, Lafayette Colorado, USA) using Lipofectamine RNAimax (Invitrogen, CA, USA). The inhibitor used is a non-hydrolysable single-stranded reverse complement of miR-449a with flanking hairpin structures and it acts by irreversible binding to the mature form of miR-449a thereby preventing its binding to its specific target. After 48 h, cells were harvested and subjected to RT-PCR and Western Blot analyses as described below. In order to study insulin signalling, cells transfected as above were treated with insulin (100 nM) and incubated for 20 min.

### Quantitative RT-PCR (qRT- PCR)

Total RNA from differentiated C2C12 cells incubated in the presence of the scramble or miR-449a or miR-449a plus inhibitor, was isolated using Trizol. 1 μg of total RNA was reverse transcribed and quantitative PCR was performed in a 10 μl reaction volume using SYBR Green PCR Master mix (Applied Biosystem) for Jag1, Hes1 and Hey1 according to the manufacturer’s recommended protocol. Primers are shown in Table 1. Also, total RNA (1 μg) from undifferentiated and differentiated C2C12 cells was reverse transcribed and the endogenous levels of miR-449a were estimated by qRT-PCR using stem loop primers [13]. The specificity of the amplified products was assessed by the dissociation curve analysis. Data was analysed and relative transcript levels were calculated by the ΔΔCt method.

**Table 1:** Sequences of the primers

Gene name	Forward Primer (5′-3′)	Reverse Primer (5′-3′)
Jag1	TGCCTCTGTGAGACCAACTG	AGGGGTCAGAGAGACAAGCA
Hes1	GTCTACACCAGCAACAGTGG	CGTCAGAAGAGAGAGGTGGG
Hey1	TCAGAGCAGTGAGGTGAAGG	AGTGCAGGCAAGGTCTACAT
Jag1 3’UTR	AATTAATTGTGTGAAGTTGGAAGCA	TGTTCCATGTTTTCATACAAAAAATT
Mutant 3’UTR	ACTTTGATTTCCTCACTTAAGGCAGGTCAGGTACTCTACGGCAAATCTAAACAGTG	CACTGTTTAGATTTGCCGTAGAGTACCTGACCTGCCTTAAGTGAGGAAATCAAAGT

### Western blot

Cells transfected as above were washed twice with ice-cold 1X phosphate-buffered saline and centrifuged at 5000 rpm at 4 °C for 10 min. Cell pellets were lysed using RIPA lysis buffer (Sigma, St. Louis, MO, USA) containing protease inhibitors (Calbiochem, Darmstadt, Germany) and lysates (40 μg) were resolved on SDS-PAGE, transferred to nitrocellulose membranes, and probed with antibodies against Jag1, p-AKT, AKT, p-PI3K, PI3K (Cell Signaling, MA, USA). Subsequent detection was with HRP-linked appropriate secondary antibodies (Bangalore Genei, India), followed by detection with the ECL Western blotting kit (GBiosciences, MO, USA). HSC70 and β-actin were used as loading controls.

### Cloning, mutagenesis, and luciferase assays

Luciferase reporter constructs harboring the 3′ untranslated region (UTR) of the mouse Jag1 gene spanning the binding sites for miR-449a were generated downstream of Renilla luciferase in a psiCheck2 vector (Promega Corporation, Madison, WI) using primers as in Table 1. Mutations in the binding site were generated using specific primers (Table 1) and a site-directed mutagenesis kit (Agilent Technologies, Santa Clara, CA). C2C12 cells were co-transfected with either the wildtype or mutated 3′ UTR reporter plasmids along with miR-449a mimics (10 nM) and/or miR-449a hairpin inhibitors (10 nM). A dual luciferase assay (Promega Corporation, MD, USA) was performed after 48 h of transfection, and luminescence was measured on an Infinite M200 Pro Multimode Reader (TECAN, Männedorf, Switzerland). Renilla luciferase values were normalized to those of firefly luciferase.

### Glucose uptake

C2C12 cells, cultured on 12-well plates, were transfected with either the scramble or the miR-449a mimic (10 nM) with or without the miR-449a inhibitor (10 nM) as described above. After 48 h, cells were washed in PBS and then incubated in Kreb’s Ringer Bicarbonate Buffer (140 mM NaCl, 5 mM KCl, 1 mM MgCl_2_, 1 mM NaH_2_ PO_4_, 2.5 mM CaCl_2_, 24 mM NaHCO_3_, 10 mM HEPES, 1% BSA) for 2 h. Cells were then pre-treated with insulin (100 nM) for 15 min and incubated in 2-NBDG (500 μM, (2-(*N*-(7-Nitrobenz-2-oxa-1,3-diazol-4-yl)Amino)-2-Deoxyglucose; Cayman Chemicals, Michigan, USA)) for 1 h in the presence of insulin (100 nM). Upon termination of incubation, cells were trypsinized and glucose uptake was measured in a fluorescence microplate reader (TECAN, Mannedorf, Switzerland). Results were normalized to the total protein content.

### Immunofluorescence

Differentiated C2C12 myotubes were seeded in six well plates (Corning CellBIND, NY, USA) and transfected with either the scramble or miR-449a mimic (10 nM) and/or the inhibitor (10 nM) for 48 h. Cells were washed with 0.1 M PBS and fixed with 4% paraformaldehyde in PBS for 30 mins. They were then permeabilized with Triton-X 100 in PBS for 10 mins. For immunodetection, cells were blocked with 1% BSA and then incubated with Notch Intracellular domain antibody (Sigma Chemical Co., St. Louis, MO, USA) at a dilution of 1:200 overnight at 4 °C and then incubated with Alexa-fluor 546 conjugated anti-rabbit secondary antibody (1:750, Invitrogen, CA, USA) along with Alexa fluor 488 Phalloidin (1,500, Invitrogen, CA, USA) stain for 1 h at room temperature. Cells were counterstained with DAPI (GBiosciences, MO, USA) for nuclear staining. Images were acquired using a High content microscope (INCell 6000, GE healthcare, PA, USA).

### Human primary skeletal muscle cell culture

Human primary skeletal muscle cells were purchased from Promocell (GmbH, Germany) and grown on 12-well plates (Corning CellBIND, NY, USA) in Skeletal muscle cell growth medium (Promocell, GmbH, Germany) according to manufacturer’s instructions. On attaining confluence, cells were transfected with either the scramble or the miR-449a mimic (10 nM) with or without its inhibitor (10 nM). On termination of incubation, cells were lysed using RIPA lysis buffer and subjected to Western blot using anti-Jag1 antibody as above. To observe the effect of miR-449a on Notch signalling pathway, immunofluorescence was performed using Notch Intracellular domain antibody (Sigma Chemical Co., St. Louis, MO, USA) as described above. Images were acquired using DeltaVision microscope (GE healthcare, PA, USA).

### Animal experiments

Ten week old male normal (C57BL/KsJ-lepr db/+) and diabetic (C57BL/KsJ lepr db/db) mice were obtained from the Animal House Facility of the CSIR-Central Drug Research Institute, Lucknow, India. Normal (weighing 22.95 ± 1.67 g with blood glucose levels: 107 ± 4 mg/dl) and diabetic animals (weighing 44.7 ± 3.87 g with blood glucose levels: 471.25 ± 128.7 mg/dl) were maintained at a 12:12 h light-dark cycle at the CSIR-Institute of Genomics and Integrative Biology, New Delhi (India) and were given ad libitum access to food and water. Experiments were performed according to the guidelines of the Institutional Animal Ethical Committee (IAEC) of the CSIR-Institute of Genomics and Integrative Biology, New Delhi, India. The gastrocnemius skeletal muscles were dissected and stored at − 80 °C until the evaluation of Jag1 protein levels by western blot analysis. Briefly 40 μg protein from skeletal muscle of normal and db/db mice were separated on SDS-PAGE, transferred to nitro-cellulose membranes and probed with anti-Jag1 antibody. HSC70 was used as the loading control.

### Densitometric analysis

AlphaEaseFC Imaging Analysis software (Alpha Innotech Corporation, San Leandro, CA) was used to evaluate protein expression by densitometric analysis.

### Statistical analysis

All experiments were performed in triplicate and statistical significance was calculated by the student’s t-test. A value of at least *p* < 0.05 was considered as statistically significant.

## Results

### miR-449a target prediction

**Fig. 1:**
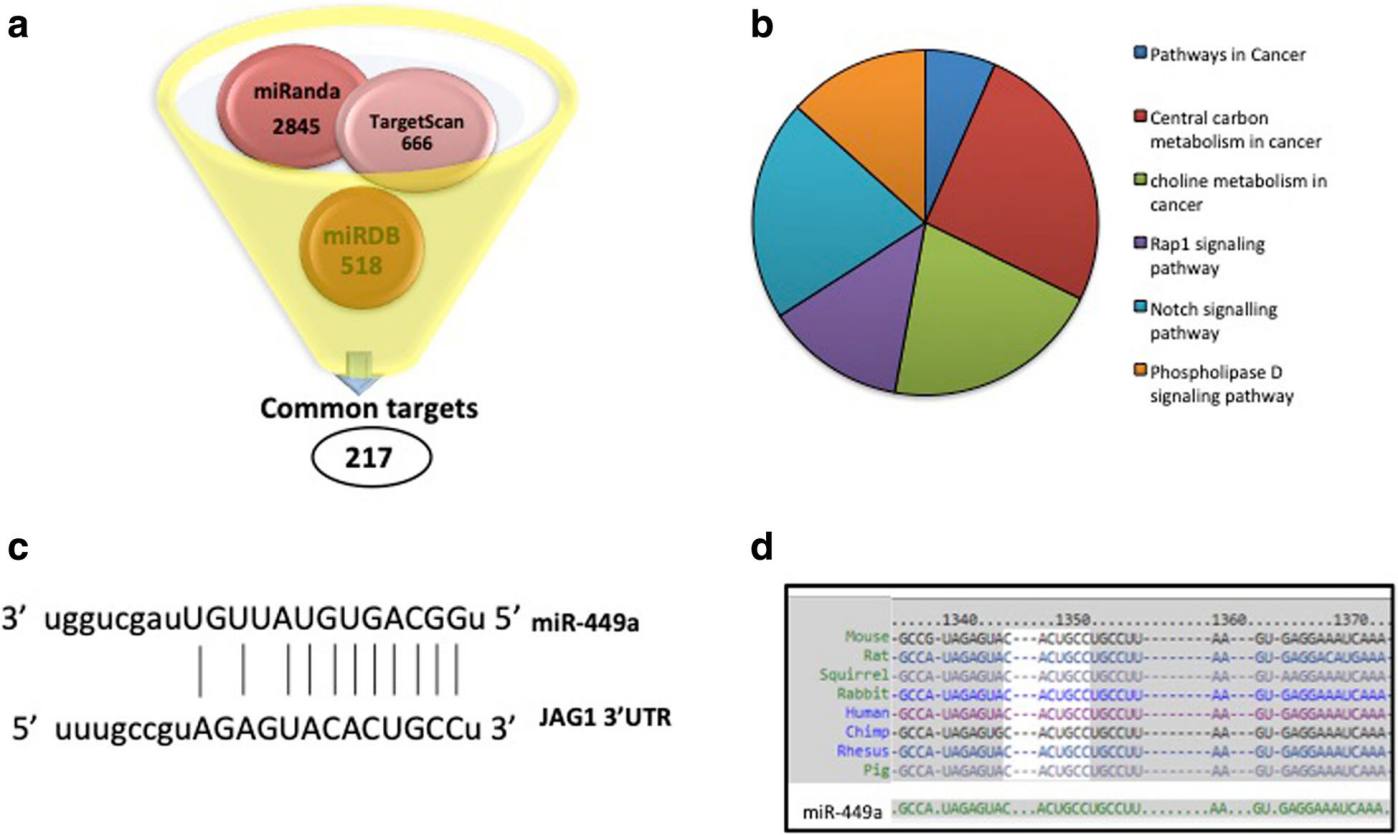
Target prediction of miR-449a. **a** Predicted miR-449a targets were extracted from miRanda, miRDB and TargetScan, and a list of 217 genes was identified as the common target set. **b** Pathway analyses of the- common predicted targets showed Notch signalling as one of the most significant over-represented pathway. The figure represents the combined scores (obtained from ENRICHR) of the pathways listed. **c** miR-449a harbors a binding site on the Jag1 3′UTR that spans across 1333–1354 nucleotides and this is conserved across several species (**d**)

Three target prediction tools were used to extract potential targets genes of miR-449a: Targetscan, miRanda and miRDB. These identified 217 common putative target genes as shown in Fig. 1a. Pathway analyses of these common predicted targets using the KEGG database within Enrichr identified Notch signalling pathway as one of the most significantly over-represented pathway (corrected *p* value = 0.0067) with the target genes being JAG1, NOTCH1, NOTCH2, DLL1 and NUMBL (Fig. 1b). The core Notch pathway operates in vastly diverse developmental and disease contexts, from stem cell regulation and heart morphogenesis to cancers and cardiomyopathies. Notch1 and DLL1 of this pathway have been validated as bonafide targets of miR-449a in celiac small intestine [20] and human airway epithelial multiciliated cells [21], respectively. Jag1 harbors a specific binding site for miR-449a in its 3’UTR (Fig. 1c) and this interaction is conserved across species (Fig. 1d) which justifies that it might be targeted by miR-449a. The functional relevance of the Notch signalling pathway in skeletal muscle metabolism in the context of diabetes has been sparsely explored. We, therefore, sought to validate the interaction between Jag1 and miR-449a and the role of this interaction in modulating Notch signalling and skeletal muscle metabolism during diabetes.

### miR-449a targets Jag1

**Fig. 2:**
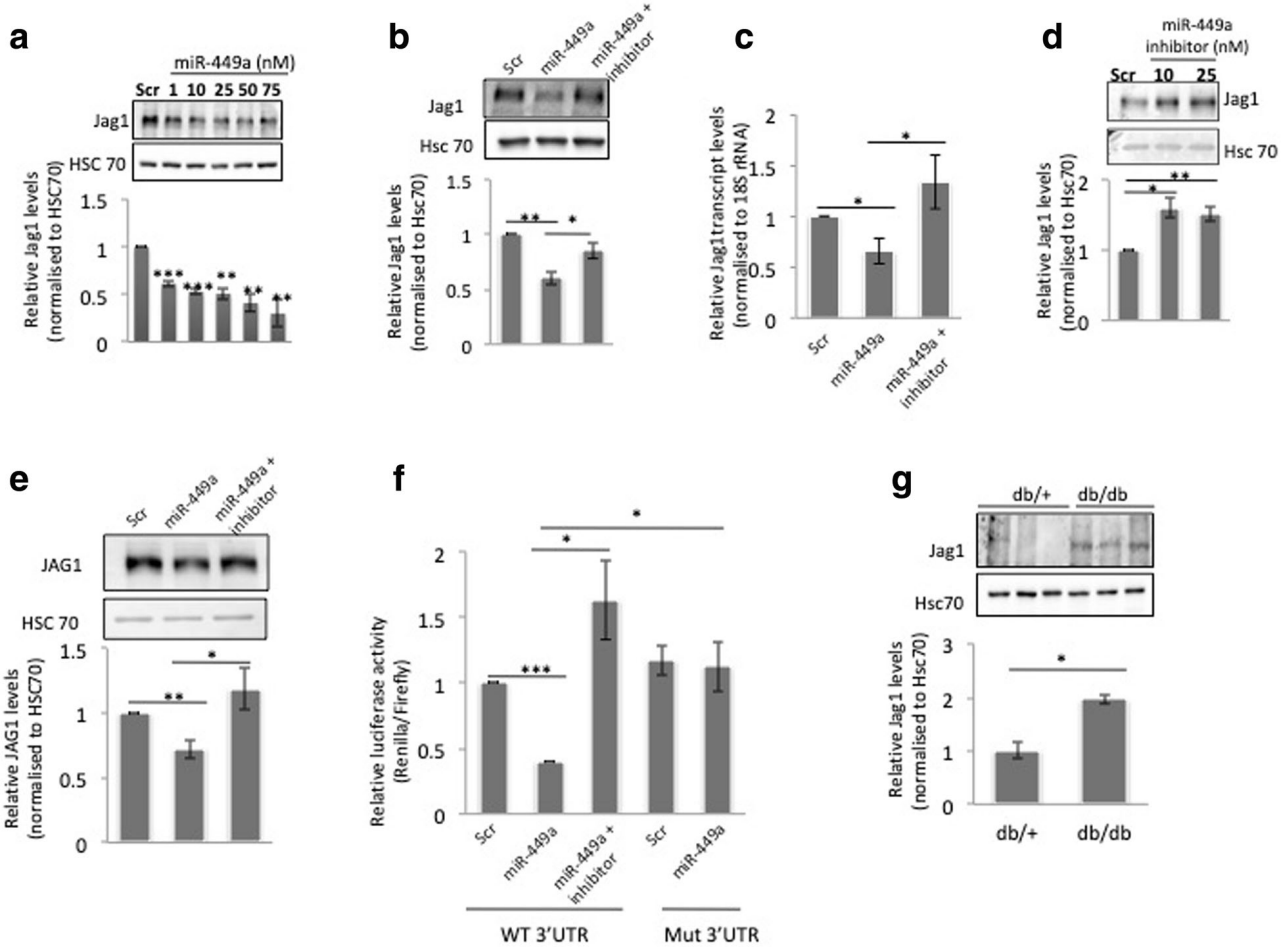
miR-449a targets Jag1 by binding to its 3’UTR. **a** Differentiated C2C12 cells were transfected with either the scramble (Scr) or the miR-449a mimic (1–75 nM). On termination of incubation (48 h), cells were lysed and 40 μg protein was resolved on SDS-PAGE and subjected to Western blot analysis using anti-Jag1 antibody. Hsc70 was taken as the loading control. **b** Differentiated C2C12 cells were incubated with miR-449a mimic (10 nM) alone or together with its inhibitor (10 nM). Control cells were transfected with scramble (Scr). After 48 h, Jag1 protein levels were assessed by Western blot analysis and Hsc70 was taken as the loading control. **c** C2C12 cells incubated as in (**b**) were assessed for the transcript levels of Jag1 by qRT-PCR. 18S rRNA was used as the normalisation control. **d** C2C12 cells were transfected with either scramble (Scr) or the miR-449a inhibitor (10, 25 nM) and incubated for 48 h. Protein levels of Jag1 were evaluated by western blot analysis and Hsc70 was taken as loading control. **e** Human primary skeletal muscle cells were transfected with either the scramble (Scr) or the miR-449a mimic (10 nM) alone or with its inhibitor and western blot analysis was performed using anti-Jag1 antibody. HSC70 was used as the loading control. **f** C2C12 cells were plated in 12-well plates and transfected with the wild-type (WT) or the mutated (mut) Jag1 3′ UTR (100 ng) together with the miR-449a mimic (10 nM) and/or its inhibitor (10 nM). Control cells were transfected with the scramble (Scr). After 48 h, cells were lysed and luciferase activity was measured as described in the ‘Materials and methods’. Firefly luciferase values were normalised to the values of Renilla luciferase. **g** The protein expression of Jag1 was assessed in db/+ and db/db mice by western blot analysis. Briefly, 40 μg protein from skeletal muscle of db/+ and db/db mice were separated on SDS-PAGE, transferred to nitro-cellulose membranes and probed with anti-Jag1 antibody. Hsc70 was used as the loading control. Densitometric analysis is given along with the respective blots. All experiments were done at least thrice and values present are mean ± SEM. ***p < 0.001, **p < 0.01, *p < 0.05

To evaluate the effects of miR-449a, C2C12 cells were transfected with either the scramble (negative control) or the miR-449a mimic in a dose dependent manner (1–75 nM). As compared to scramble, there was a significant decrease in the protein levels of Jag1 (Fig. 2a). To check the specificity of this observation, cells were transfected with the mimic alone or together with its inhibitor. As shown in Fig. 2b and c, while miR-449a significantly decreased Jag1 both at the protein and transcript levels, this was significantly prevented in the presence of the miR-449a inhibitor. On the contrary, in cells transfected with the miR-449a inhibitor alone, cellular levels of Jag1 significantly increased at the doses 10 and 25 nM (Fig. 2d). All these suggest that miR-449a possibly targets Jag1 and decreases its levels. This was also validated in primary human skeletal muscle cells and as shown in Fig. 2e, miR-449a significantly decreased Jag1 protein levels and this was prevented in the presence of the miR-449a inhibitor. In order to validate the specific binding of miR-449a on the 3’UTR of Jag1 transcript, dual luciferase assay was performed. Luciferase reporter constructs of the Jag1 3’UTR harbouring the miR-449a binding site were generated and mutations at 4 nucleotides at the binding site were created using specific primers (Table 1). C2C12 cells were co-transfected with either the wildtype or mutated 3’UTR reporter plasmids along with miR-449a mimic and/or miR-449a hairpin inhibitors. After 48 h, miR-449a decreased the luciferase activity of the reporter plasmid harboring the Jag1 3’UTR (Fig. 2f). This decrease in the luciferase activity by miR-449a was prevented, both in the presence of the miR-449a inhibitor and in the reporter construct harbouring mutations in the miR-449a binding site on the Jag1 3’UTR. All these suggest that miR-449a targets Jag1 and decreases its levels by binding to its 3’UTR. Interestingly, using quantitative RT-PCR (with miR-449a specific primers [13]), miR-449a levels were observed to be significantly decreased in differentiated C2C12 cells as compared to undifferentiated cells. Since a previous report from our laboratory demonstrated decreased levels of miR-449a in the skeletal muscle during diabetes, we sought to evaluate the levels of its target, Jag1 in these tissues. As shown in Fig. 2g, there was an increase in the protein levels of Jag1 in the skeletal muscle of diabetic mice suggesting increased Notch signalling. This corroborates with previous findings where dysregulation of Notch signalling components during diabetes has been observed in different tissues such as pancreas and liver [22–24].

### miR-449a binding to Jag1 inhibits notch target genes and NICD nuclear translocation

**Fig. 3:**
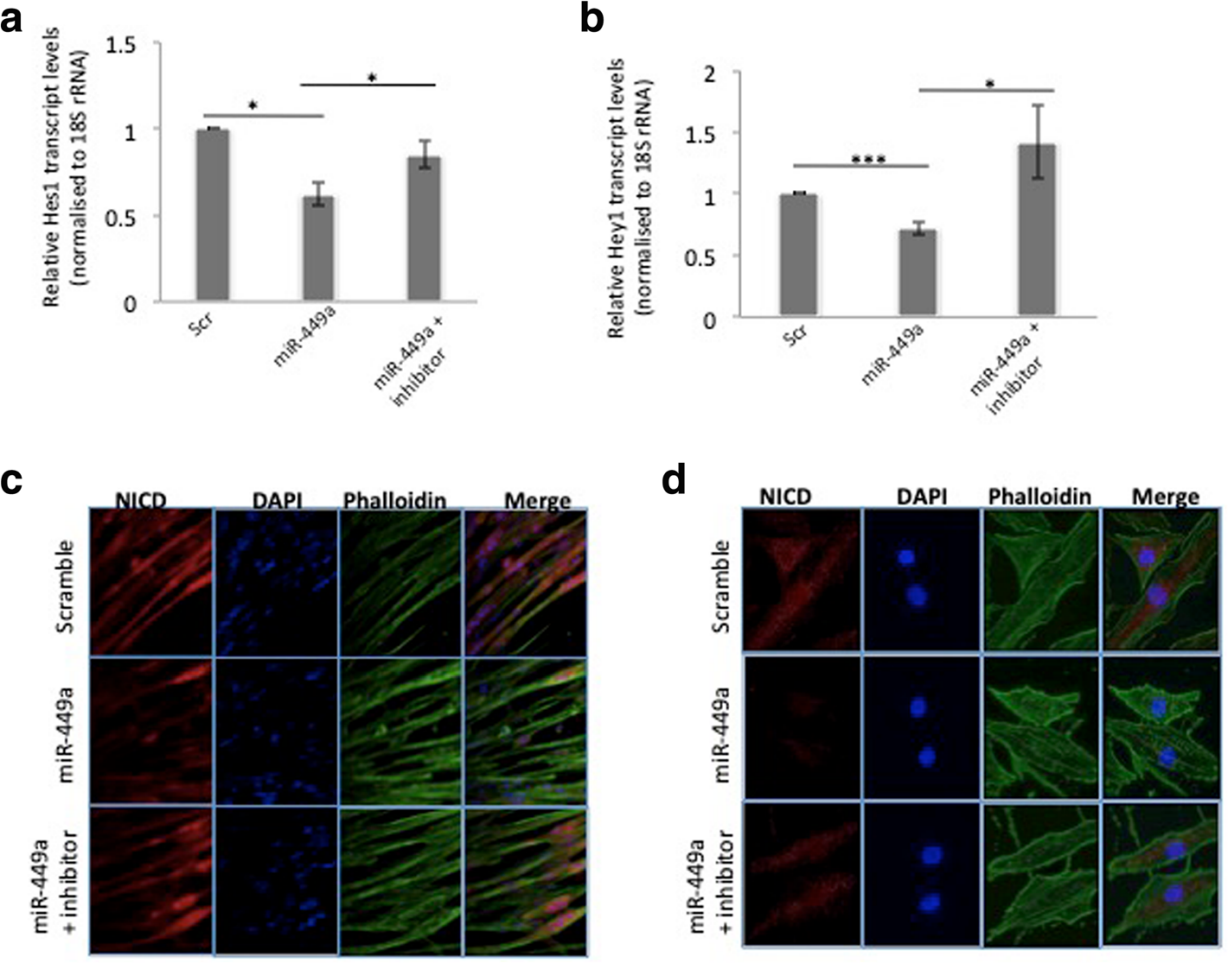
miR-449a binding to Jag1 inhibits Notch target genes and NICD levels. **a** Differentiated C2C12 cells were transfected with the miR-449a mimic (10 nM) in the absence or presence of its inhibitor (10 nM). After 48 h, cells were lysed and relative transcript levels of Notch target genes, Hes1 (**a**) and Hey1 (**b**) were quantified by qRT-PCR and normalised to 18S rRNA. Control cells were transfected with the scramble (Scr). **c** Differentiated C2C12 cells were grown on six-well plates and transfected with miR-449a (10 nM) alone or in the presence of its inhibitor (10 nM). After 48 h, cells were fixed in 4% formaldehyde and immunofluorescence was performed with anti-NICD antibody as described in ‘Materials and Methods’ (**d**) Human primary skeletal muscle cells were grown on chamber slides and transfected with either scramble (Scr) or miR-449a alone or with its inhibitor (10 nM). As in (c), immunofluorescence was performed and cells were visualised in fluorescence microscope. Incubations were repeated at least three times and values are presented as mean ± SEM. ***p < 0.001, *p < 0.05

Results until now indicate Jag1 as a specific target of miR-449a. Since Jag1 is a Notch ligand and an effector molecule of Notch signalling, we sought to assess the effect of miR-449a on the activation of this pathway. Ligand mediated activation of this pathway induces a series of proteolytic cleavages of the members of the Notch family receptors and these cleaved members called Notch Intracellular domain (NICD), stimulate the transcription of target genes namely, Hairy enhancer of split (Hes) and Hes-related (Hey) [25]. We, therefore, attempted to assess the effect of miR-449a-Jag1 interaction on the transcript levels of Hes1 and Hey1. While miR-449a significantly inhibited the transcript levels of Hes1 and Hey1, this decrease was prevented in the presence of the inhibitor of miR-449a (Fig. 3a, b). The expression of cleaved Notch1 (NICD) was confirmed by immunofluorescence. As shown in Fig. 3c, staining for NICD was significantly reduced in miR-449a transfected C2C12 cells and this inhibition was prevented when cells were transfected in the presence of miR-449a inhibitor. This was further confirmed in human primary skeletal muscle cells as shown in Fig. 3d. High intensity of NICD staining was seen in scramble transfected cells while the expression of NICD was drastically decreased in miR-449a transfected cells and this decrease was prevented in the presence of its inhibitor. These data indicate that miR-449a binding to Jag1 inhibits the activation of the Notch signalling pathway and hence its target genes, Hes1 and Hey1.

### miR-449a activates the insulin signalling pathway

**Fig. 4:**
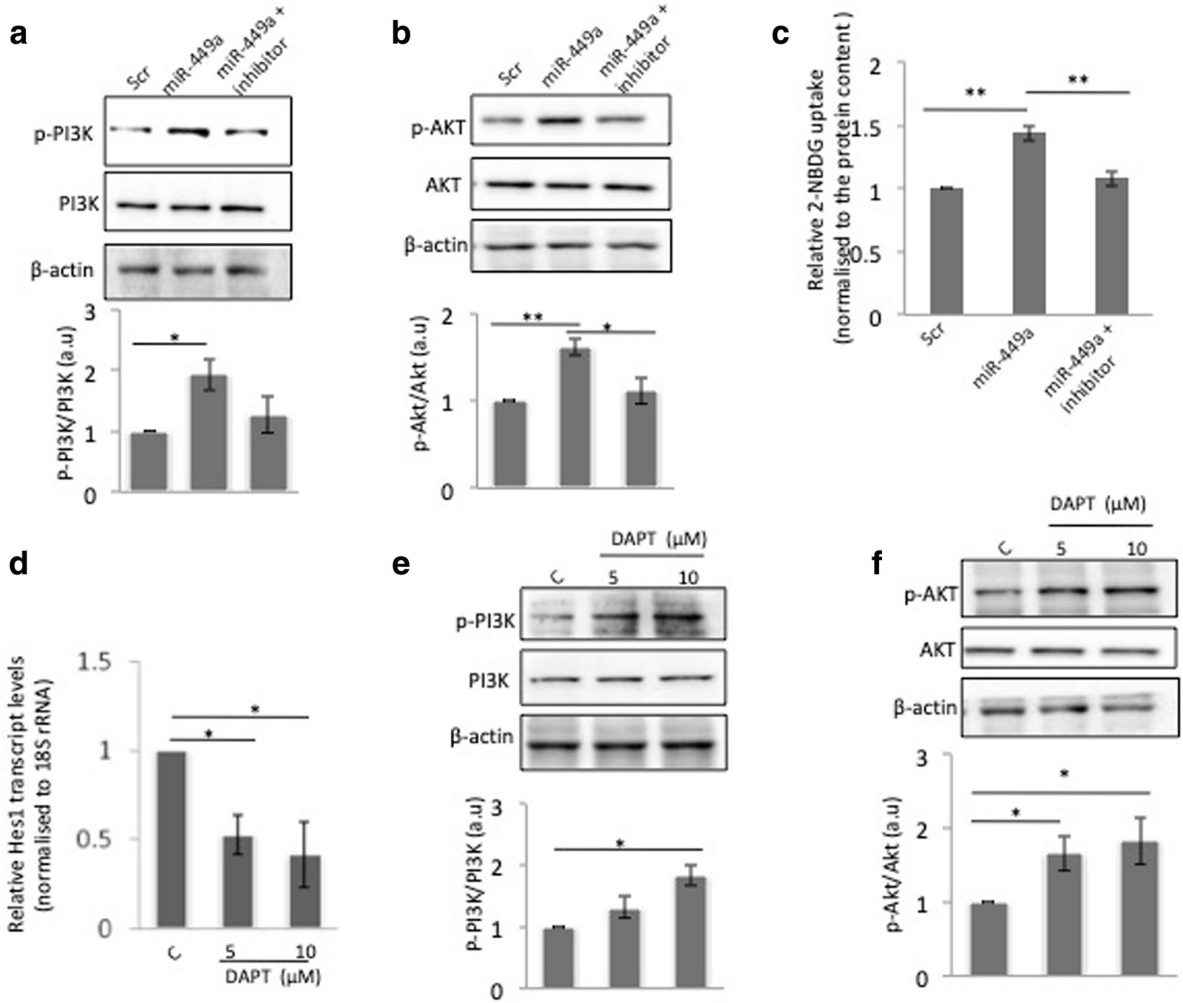
Overexpression of miR-449a and inhibition of Notch signalling promote insulin signalling. **a** Differentiated C2C12 cells were transfected with the miR-449a mimic (10 nM) alone or together with its inhibitor (10 nM). Control cells were transfected with the scramble (Scr). After 48 h, cells of all groups were treated with insulin (100 nM) for 20 min and on termination of incubation, they were lysed and probed for the levels of p-PI3K, PI3K (**a**), p-AKT, AKT (**b**) by western blot analysis. β-actin was used as loading control. **c** C2C12 cells were transfected with either the scramble or miR-449a alone or together with its inhibitor and after 48 h, were incubated in Kreb’s-Ringer bicarbonate buffer for 2 h. Cell were then pre-incubated with insulin (100 nM) for 15 min, followed by incubation with 2-NBDG (500 μM) in the presence of insulin (100 nM) for 1 h. On termination of incubation, fluorescence intensity of 2-NBDG was measured as described in ‘Materials and Methods’ section. **d** Differentiated C2C12 cells were incubated with the Notch inhibitor, DAPT (5,10 μM) for 24 h and the transcript levels of Notch target gene, Hes1 were quantified by qRT-PCR. Control cells were incubated in the presence of DMSO (C). 18S rRNA was used as loading control. In another experiment, cells were incubated as in (**d**) for 24 h and then incubated with insulin (100 nM) for 20 min and evaluated for the levels of p-PI3K, PI3K (**e**) and p-AKT,AKT (**f**). β-actin was used as the loading control. Densitometric analyses are given below the respective blots. All the experiments were done at least thrice and data presented as mean ± SEM. *p < 0.05, **p < 0.01

Results from the above suggest that by inhibiting Jag1, miR-449a inhibits the Notch signalling pathway. In order to examine the effect of miR-449a on insulin signalling in skeletal muscle cells, we transfected C2C12 cells with either the scramble or with miR-449a or with miR-449a together with its inhibitor and after 48 h, cells were incubated in the presence of insulin (100 nM) for 20 min. As compared to scramble transfected cells, overexpression of miR-449a in C2C12 cells elevated the levels of insulin stimulated PI3K and AKT phosphorylation with no change in their total levels. Also, such increase was prevented in cells transfected with the miR-449a inhibitor (Fig. 4a, b), suggesting that miR-449a activates insulin signalling in these cells. This was also validated by studying insulin stimulated glucose uptake in C2C12 cells and as shown in Fig. 4c, miR-449a caused a significant increase in insulin stimulated glucose uptake as compared to scramble transfected cells. This increase was prevented in the presence of the miR-449a inhibitor. All these suggest that miR-449a increases insulin signalling in these cells.

### Notch signalling inhibition promotes activation of the insulin signalling pathway

Results until now establish that miR-449a inhibits the Notch signalling pathway and also influences insulin signalling in C2C12 cells. We then sought to determine if inhibition of the Notch signalling pathway, by itself improves insulin signalling in these cells. We used a γ-secretase inhibitor, DAPT to inhibit the Notch Signalling pathway. Treatment of C2C12 cells with DAPT for 24 h at doses of 5 and 10 μM inhibited the Notch activity as evident by reduced transcript levels of Hes1 as shown in Fig. 4d. The inhibited Notch activity increased the levels of insulin stimulated PI3K and AKT phosphorylation as compared to control (DMSO treated (C)) cells as shown in Fig. 4e and f. Taken together, these suggest that miR-449a interacts with and inhibits Jag1 levels and thereby inhibits the Notch signalling pathway and this regulation influences the insulin signalling pathway.

## Discussion

In a previous report from our laboratory, we had shown that the intronic miRNA, miR-449a is regulated by histone acetylation/deacetylation at the promoter of its host gene, Cdc20b [13].

miR-449a is located in the second intron of Cdc20b on chromosome 5 in human and on chromosome 13 in mouse. It is classified as a member of the miR-34 family and is fairly conserved across vertebrates species. In cancer cells, miR-449a expression is epigenetically inactivated by histone trimethylation at H3K27me3, this being reversed by epigenetic drug treatment [26]. Its levels are significantly silenced by aberrant DNA methylation in medulloblastoma tumor cells [18]. Interestingly, miR-449a has been shown to target Flot2 and regulate EMT, thereby inhibiting gastric cancer invasion [27]. Noonan et al. have shown that by targeting HDAC1, miR-449a induces the cell-cycle inhibitory protein, p27 expression and promotes cell growth arrest. Such growth-supressing function of miR-449a helps in better understanding of cancer mechanisms and better prognostic evaluation [17]. miR-449a levels are decreased in hepatocellular carcinoma and its overexpression inhibits cell motility and metastasis [15]. By targeting ADAM10, miR-449a inhibits cell proliferation and invasion. Also, overexpression of ADAM10 attenuates such inhibitory effects of miR-449a [14]. Its levels are also down regulated in glioblastoma and detailed studies demonstrated that it induces apoptosis and reduces cell proliferation [16].

Our data show that miR-449a that is regulated by histone modification [13], targets the Notch ligand, Jag1 and consequently inhibits the Notch signalling pathway. Notch signalling is highly conserved across species and plays a critical role in cell fate decision and lineage restriction by interacting with neighbouring cells [28]. The signalling initiates by binding of Notch ligands namely Jag1/2 and Delta like 1/3/4 to the Notch receptor that results in secretase catalysed cleavage events resulting in transcription of Notch target genes, Hes and Hey [25, 29] . Mutations in the Notch pathway result in multiple developmental defects [30] and in some cases, deletion results in embryonic lethality [31, 32].

However, very little is known about the role and involvement of the Notch signalling pathway in cellular metabolic pathways and correlation to obesity, insulin resistance and diabetes. Notch target gene expression is increased in obese and insulin resistant mouse models [23, 33] and inhibition of this pathway in the liver resulted in decreased hepatic glucose output and lipid accumulation [34]. Also, glucose intolerance and fatty liver phenotypes were observed in animals with constitutive activation of hepatocyte Notch signalling. In morbidly obese individuals, hepatocyte Notch activation positively correlates with gluconeogenic genes’ expression and hyperglycemia [24]. Expression of Notch target genes, Hes and Hey positively associates with insulin resistance and hepatic fat content. Our data show that inhibition of Notch signalling using DAPT, a γ-secretase inhibitor, increases insulin stimulated PI3K and AKT phosphorylation suggesting that inhibited Notch signalling promotes insulin signalling in C2C12 cells. Insulin stimulated PI3K and AKT phosphorylation and glucose uptake are increased by miR-449a and this is prevented in the presence of miR-449a along with its inhibitor. While all these might suggest that the effect of miR-449a on insulin signalling might be by targeting Jag1 and consequently through inhibition of the Notch signalling pathway, these need further detailed in-depth investigation.

None-the less, our study shows that miR-449a targets Jag1 and this miR-449a-Jag1 interaction might have implications in regulating the insulin signalling pathway in the skeletal muscle. We also observed that Jag1 levels are elevated in the skeletal muscle of diabetic db/db mice and since miR-449a levels are inhibited in these animals [13], we think that such decreased miR-449a levels are responsible for increased Jag1 levels in the skeletal muscle during diabetes.

Here, we demonstrate that miR-449a targets the Notch ligand, Jag1 and in doing so, regulates the Notch signalling pathway. Such regulation of the Notch signalling pathway, in turn modulates the insulin signalling pathway and therefore, altered levels of miR-449a is critical in deregulated insulin signalling as seen during diabetes. Hence, normalising miR-449a levels might be a valuable tool to restore impaired insulin signalling and improve glucose uptake in the skeletal muscle and consequently prevent diabetic complications.

## Conclusions

In this study, we demonstrate that miR-449a regulates the levels of the Notch ligand, Jag1 by binding to its 3’UTR. In doing so, the miR-449a-Jag1 interaction inhibits the Notch signaling pathway as evident by inhibition of NICD (notch intracellular domain) levels and also those of Notch target genes, Hes1 and Hey1. Both miR-449a increase and Notch signalling inhibition significantly activated insulin stimulated PI3K and AKT phosphorylation. Results suggest that miR-449a-Jag1 interaction is critical in Notch signalling and insulin signalling in the skeletal muscle and modulating this interaction might hold therapeutic potential for impaired skeletal muscle metabolism during diabetes.

## Data Availability

All data generated in this study are included in the manuscript.

## Abbreviations

BSA: Bovine Serum Albumin
DAPT: **N**-[N-(3,5-Difluorophenacetyl)-L-alanyl]-S-phenylglycine t-butyl ester
DMEM: Dulbecco’s modified Eagle’s medium
DMSO: Dimethyl sulfoxide
ECL: Enhanced chemiluminescence
EMT: Epithelial-Mesenchymal transition
HDAC: Histone Deacetylases
HES: Hairy enhancer of split
HEY: Hes related with YRPW motif protein
HRP: horseradish peroxidise
JAG: Jagged
MET: Mesenchymal Epithelial Transition
NICD: Notch Intracellular Domain
PBS: Phosphate Buffer Saline
PI3K: Phosphoinositide 3-kinase
